# Central Disorders of Hypersomnolence, Restless Legs Syndrome, and Surgery With General Anesthesia: Patient Perceptions

**DOI:** 10.3389/fnhum.2018.00099

**Published:** 2018-03-20

**Authors:** Vincent LaBarbera, Paul S. García, Donald L. Bliwise, Lynn M. Trotti

**Affiliations:** ^1^Sleep Center and Department of Neurology, Emory University School of Medicine, Atlanta, GA, United States; ^2^Department of Anesthesiology, Emory University School of Medicine, Atlanta, GA, United States; ^3^Research and Anesthesiology Service Lines, Atlanta VA Medical Center, Atlanta, GA, United States

**Keywords:** general anesthesia, idiopathic hypersomnia, narcolepsy, restless legs syndrome, excessive daytime sleepiness

## Abstract

**Introduction:** The importance of obstructive sleep apnea in patients undergoing surgery with general anesthesia is well-defined, but the surgical and anesthetic implications of other sleep disorders are less clear. We sought to evaluate response to surgery with general anesthesia in patients with central disorders of hypersomnolence or restless legs syndrome.

**Methods:** We surveyed patients on their most recent surgical procedure with general anesthesia, querying about procedure, recovery, and any changes in sleep disorder symptomatology following the procedure.

**Results:** Forty-five patients with restless legs syndrome and 57 patients with central disorders of hypersomnolence (15 narcolepsy type 2, 1 narcolepsy type 1, 30 idiopathic hypersomnia, 1 Kleine-Levin syndrome, and 10 subjective sleepiness) completed the survey, with response rates of 45.5 and 53.8%, respectively. While patients in both groups were equally likely to report surgical complications and difficulty awakening from anesthesia, hypersomnolent patients were more likely to report worsened sleepiness (40% of the hypersomnolent group vs. 11% of the RLS group, *p* = 0.001) and worsening of their sleep disorder symptoms (40% of the hypersomnolent group vs. 9% of the RLS group, *p* = 0.0001).

**Conclusion:** Patients with sleep disorders other than sleep apnea frequently report surgical or anesthetic complications. Patients with hypersomnolence disorders commonly perceive that their sleep disorder worsened following a procedure; whether this might be related to long term effects of general anesthesia in a particularly vulnerable clinical population requires further study.

## Introduction

The importance of obstructive sleep apnea for the perioperative management of surgical patients is now widely acknowledged and codified within professional guidelines (American Society of Anesthesiologists Task Force on Perioperative Management of patients with obstructive sleep apnea, 2014). In contrast, the potential importance of other sleep disorders is less clear. Concern about anesthetic-induced worsening of restless legs syndrome (RLS), a sensorimotor sleep disorder resulting in nocturnal leg discomfort and sleep disruption, was raised in a prospective series that found an RLS incidence of 9% in patients undergoing spinal anesthesia (Högl et al., 2002). This finding was not replicated in a second study (Crozier et al., 2008), albeit with a study population with very different composition. The perioperative period may be a time of heightened vulnerability to worsening RLS symptoms, because of prolonged immobility, blood loss, iatrogenic sleep loss, temporary cessation of symptomatic treatment, and use of medications such as metoclopramide that can worsen RLS (Högl et al., 2012; Goldstein, 2015). However, the possibility of acute or chronic effects of general anesthesia has not been well-defined in RLS patients.

Similarly, relatively little study has been directed at anesthetic implications for patients with central disorders of hypersomnolence. This group of disorders, which includes narcolepsy type 1 (with cataplexy), narcolepsy type 2 (without cataplexy), and idiopathic hypersomnia, results in persistent and pathologic daytime sleepiness despite adequate or prolonged amounts and quality of nocturnal sleep. Kleine-Levin syndrome, also a central disorder of hypersomnolence, refers to a syndrome consisting of bouts of recurrent hypersomnia separated by periods of relative normalcy. Potential concerns for the perioperative management of patients with central disorders of hypersomnolence raised by case reports and small case series include: (1) the appearance of peri-operative cataplexy, sleepiness, or other disease-specific symptoms; (2) the chronic use of psychotropic and/or vasoactive medications, including amphetamines, in patients with these disorders; and (3) delayed emergence from anesthesia (Mesa et al., 2000; Peláez et al., 2004; Burrow et al., 2005; Fischer et al., 2006; Ozkose et al., 2007; Staikou et al., 2007; Doyle and Wilkinson, 2008; Dahaba et al., 2009; Morimoto et al., 2011; Stoicea et al., 2014; Tzabazis et al., 2015; Aflaki et al., 2017). The concern regarding delayed emergence may be particularly relevant to a subgroup of patients with central disorders of hypersomnolence who demonstrate the presence of a positive allosteric modulator of gamma-aminobutyric acid Type A (GABA-A) receptors within spinal fluid (Rye et al., 2012). GABA is known to be a key player in sleep onset (Lu and Greco, 2006; Franks and Zecharia, 2011) and sleepiness in these disorders can be reversed using negative allosteric modulators of GABA-A receptors such as flumazenil and clarithromycin (Trotti et al., 2015, 2016). Importantly, the GABA-A receptor is also of major importance in the pharmacology of anesthesia, with increased inhibitory signaling via agent-specific binding sites being the mechanism by which most commonly used pharmacologic agents (i.e., isoflurane, sevoflurane, desflurane, propofol, and etomidate) produce general anesthesia (Garcia et al., 2010).

A recent, controlled, retrospective study of patients with narcolepsy, primarily type 2, who underwent general anesthesia reported similar intraoperative and phase 1 anesthetic recovery among patients with narcolepsy and controls (Cavalcante et al., 2017). However, it identified an increased risk of emergency response team activation during post-operative hospitalization among those with narcolepsy. Anecdotally, we have noted that a seemingly large proportion of our hypersomnolent patients report complicated recoveries from surgeries involving general anesthesia. To expand upon the existing literature regarding anesthetic response in patients with non-apneic sleep disorders, we performed a pilot study, systematically assessing patients' perceptions of their surgical/anesthetic recovery and post-operative course in those with central disorders of hypersomnolence and restless legs syndrome.

## Methods

Potential subjects were identified from existing research registries. For the hypersomnolent group, we screened all enrolled patients for a history of surgery with general anesthesia, first by chart review and then via telephone interview. As the RLS registry is larger, having been established more than 10 years ago, we screened only those patients in the RLS registry who had been evaluated at the Sleep Center in the 18 months prior to the study, to obtain a similar group size. The RLS patients were similarly screened for a history of surgery with general anesthesia.

Patients underwent comprehensive sleep medicine evaluation at the Emory Sleep Center, a tertiary referral center. Patients with central disorders of hypersomnolence were studied with overnight polysomnography followed by multiple sleep latency test and classified as having narcolepsy type 1 or 2, idiopathic hypersomnia, or Kleine-Levin syndrome (2014). Cases meeting clinical but not electro-physiologic criteria were classified as having subjective excessive daytime sleepiness (sEDS) (Pizza et al., 2013). Hypersomnolent patients are routinely offered diagnostic lumbar puncture as part of their evaluation and were considered eligible for inclusion in the present study if their CSF demonstrated *in vitro* potentiation of GABA-A receptors >60% (Rye et al., 2012). Patients with restless legs syndrome were diagnosed based on face-to-face interview conforming to criteria of the International Restless Legs Syndrome Study Group (Allen et al., 2003, 2014). Patients with both RLS and a central disorder of hypersomnolence were excluded.

Identified patients with a history of general anesthesia were sent an online survey regarding daytime sleepiness, restless legs syndrome symptoms, sleep habits, and anesthesia history. The survey program (REDCap) employed branching logic. We recorded patient characteristics and experience with most recent surgery with general anesthesia, including perception of any operative complication, anesthetic complication, Post-anesthesia Care Unit (PACU)/recovery complication, or change in baseline symptomology following the procedure. Subjects were asked to describe their recovery room and anesthetic experiences in their own words, which were used to determine presence or absence of difficulty in awakening from anesthesia.

Outcomes of interests were the proportion of patients who felt their sleep patterns, daytime sleepiness, or primary sleep symptoms (i.e., either sleepiness or restlessness) changed after general anesthesia/surgery, and rates of perceived complications, including difficulty in awakening. Specific changes to sleep were evaluated by providing multiple choices for how symptoms may have changed.

Clinical information was compared between groups using *t*-tests (correcting for unequal variance when necessary) for continuous variables and chi-square or Fisher test for categorical variables. Symptom response to procedure/anesthesia was dichotomized as “worse” vs. “better or no change.” Statistical analyses were performed using SAS 9.3. This study was approved through the Emory University Institutional Review Board. Consent discussion was performed in-person or by phone by one of the authors (VL), then written informed consent was obtained from all subjects.

## Results

**Figure 1:**
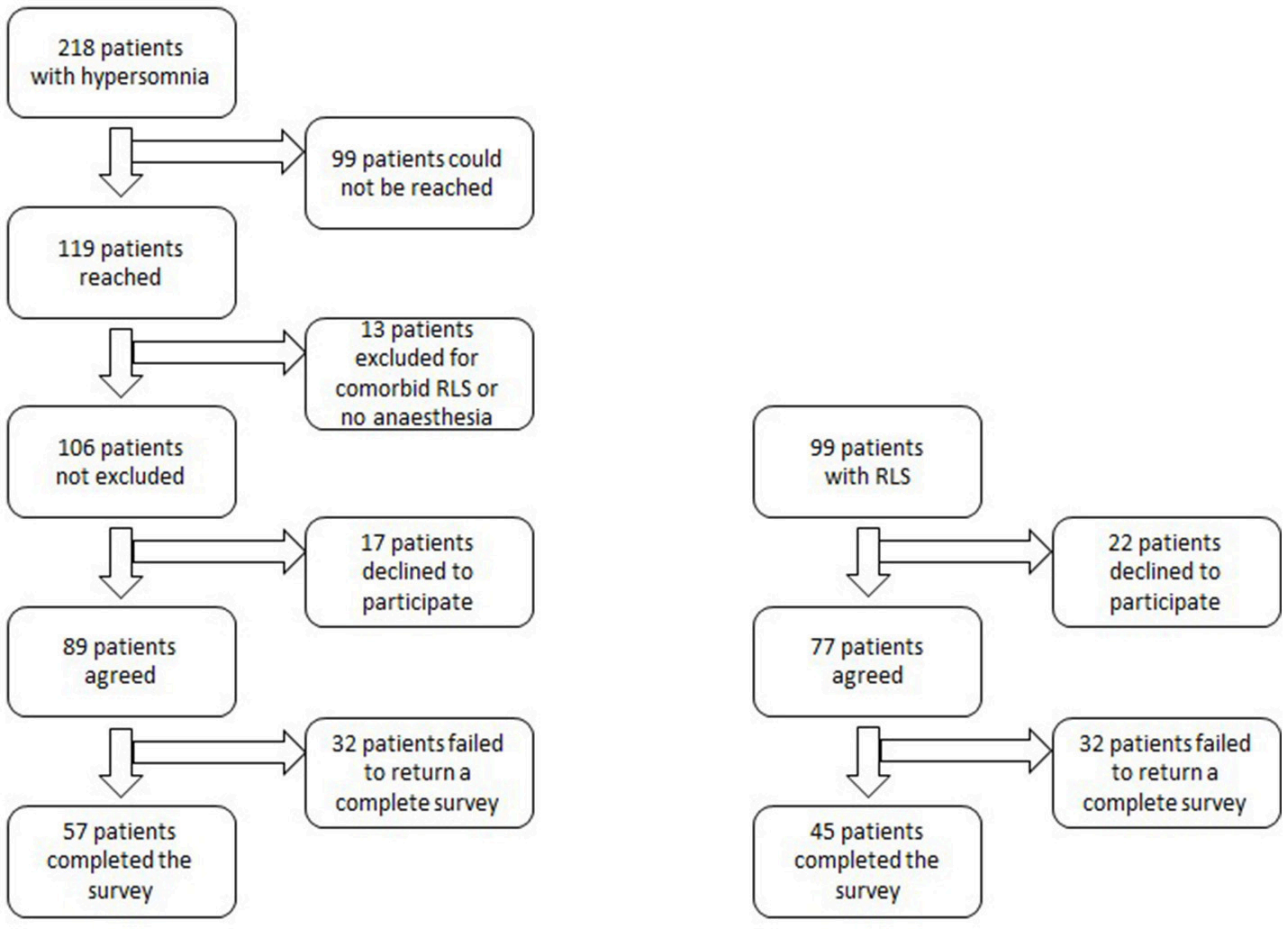
Flow chart of survey data acquisition.

For the hypersomnolent group, electronic surveys were sent to 89 patients, after excluding those who could not be reached, did not have a general anesthesia history, had comorbid restless legs syndrome, or declined to participate (Figure 1). Fifty-seven participants with hypersomnolence completed the survey (response rate 53.8%). Diagnoses of these patients included: narcolepsy type 2 (*n* = 15), narcolepsy type 1 (*n* = 1), idiopathic hypersomnia (*n* = 30), Kleine-Levin syndrome (*n* = 1), and sleepiness not meeting electro-diagnostic criteria for one of the above syndromes (sEDS, *n* = 10). Ninety-nine patients were contacted from the RLS registry. Twenty-two patients were not interested in participating. Forty-five patients completed the survey (response rate, 45.5%). Response rates between hypersomnolent and RLS patients were not significantly different (*p* = 0.23).

Study subject characteristics are detailed in Table 1. The two groups were similar with respect to gender, BMI, and proportion with a self-reported comorbid psychiatric disease. Age and proportion with comorbid medical disease burden were significantly different, with the RLS group significantly older and with more medical comorbidity (*p* < 0.0001 and 0.004, respectively). Sleep habits were different between groups as expected, with the hypersomnolence group reporting an average 8.7 ± 2.0 h per night of sleep vs. 6.2 ± 1.2 h in the RLS group (*p* < 0.0001). The Epworth Sleepiness Scale (Johns, 1991) was significantly higher in the hypersomnolent group 13.1 ± 5.7 than in the RLS group (10.9 ± 5.6, *p* = 0.046). The hypersomnolence group reported needing significantly more alarms to wake up every morning (3.5 ± 1.5 alarms, vs. 1.9 ± 1.6 in the RLS group, *p* < 0.0001). Types of surgery were not significantly different between the groups.

**Table 1:** Patient characteristics.

	**Hypersomnolence disorders (*n* = 57)**	**Restless legs syndrome (*n* = 45)**
Male gender	19 (33.3%)	18 (40.0%)
Age	39.8 (12.5)	65.0 (10.5)
BMI	27.9 (6.5)	28.4 (6.8)
Average hours sleep per night	8.7 (2.0)	6.2 (1.2)
Average awakenings per night	1.4 (1.8)	3.0 (2.1)
Subjective sleep quality	3.4 (1.3)	3.9 (0.9)
# alarms (or snoozes) needed to wake up^*^	3.5 (1.5)	1.9 (1.6)
Epworth score	13.1 (5.7)	10.9 (5.6)
N with medical comorbidities	33 (57.9%)	38 (84.4%)
N with psychiatric comorbidities	24 (42.1%)	15 (33.3%)
Time between most recent surgery and questionnaire completion, years	8.4 (7.3)	6.4 (8.3)
Type of surgery	1:18 (31.6%) 2:0 3:21 (36.8%) 4:8 (14.0%) 5:2 (3.5%) 6:7 (12.3%) 7:1 (1.8%)	1:6 (13.3%) 2:1 (2.2%) 3:17 (37.8%) 4:4 (8.9%) 5:3 (6.7%) 6:10 (22.2%) 7:4 (8.9%)

*Values are reported as mean (standard deviation) or number (percent). BMI, body mass index; Subjective sleep quality is measured on a 1–6 scale, where higher is worse; Types of surgeries are: 1, Breast, plastics, and dental; 2, Open abdominal procedures; 3, Laparoscopic abdominal procedures, gynecological, and urological procedures; 4, Otolaryngological and Oromaxillary facial surgeries; 5, Neurosurgery and Orthopedic spine procedures; 6, Orthopedic and podiatric procedures; 7, Cardiothoracic surgery*.

* *did not differentiate weekends vs. weekdays*.

The two groups of patients were no different in terms of the percentage reporting complications related to surgery or anesthesia. Surgical complications were noted by 11.1% of hypersomnolent patients and 4.4% of RLS patients (*p* = 0.29), recovery complication by 28.3% of hypersomnolent patients and 15.9% of RLS patients (*p* = 0.15), and abnormal anesthetic reaction by 25% of hypersomnolent patients and 15.6% of RLS patients (*p* = 0.25). In the hypersomnolent group, 10 patients (17.5%) specifically mentioned difficulty in awakening from their procedure or anesthesia, similar to the 7 patients (15.6%) in the RLS group (*p* = 0.79).

Nineteen (33.3%) of the hypersomnolent patients endorsed worsened sleep patterns following their most recent procedure, and 23 (40.4%) endorsed worsened daytime sleepiness. Five (11.1%) of the RLS participants endorsed worsened sleep patterns and five reported worsened daytime sleepiness. Both were significantly different between the two groups (*p*-values 0.009 and 0.001, respectively, see Table 2). In analysis of 24 people who endorsed worsened sleep patterns, hypersomnolent patients were much more likely to report sleeping more hours [14 (73.7%) of the hypersomnolent patients vs. 0 (0%) of the RLS patients, *p* = 0.006], and RLS patients were much more likely to report sleeping fewer hours [4 (80%) of the RLS patients vs. 2 (10.5%) of the hypersomnolent patients, *p* = 0.007].

**Table 2:** Outcomes of most recent surgery with general anesthesia.

	**Hypersomnolence disorders (*n* = 57)**	**Restless legs syndrome (*n* = 45)**	***p*-value**
Surgical complications	6 (11.1%)	2 (4.4%)	0.29
Recovery complications	15 (28.3%)	7 (15.9%)	0.15
Abnormal reaction to anesthesia	13 (25.0%)	7 (15.6%)	0.25
Slow to awaken from anesthesia	10 (17.5%)	7 (15.6%)	0.79
Worsened sleep patterns/habits	19 (33.3%)	5 (11.1%)	0.009
Worsened daytime sleepiness	23 (40.4%)	5 (11.1%)	0.001

*Values are reported as number (percent)*.

Among the 28 people who endorsed worsening sleepiness, hypersomnolent patients were much more likely to report worsening difficulty awakening in the morning [18 (78.3%) of the hypersomnolent patients vs. 1 (20%) of the RLS patients, *p* = 0.03] and increasing limitations to daily activities from sleepiness [18 (78.3%) of the hypersomnolent patients vs. 1 (20%) of the RLS patients, *p* = 0.03].

Considering the effect of surgery with general anesthesia on the patients' primary sleep problem (i.e., change in sleepiness in the hypersomnolent group vs. change in restlessness in the RLS group), the two groups were significantly different. Patients with hypersomnolence were much more likely to report worsening of sleepiness than patients with RLS were to report worsening of RLS symptoms [hypersomnolent patients reported themselves as better in 1 (1.75%), unchanged in 33 (57.9%), and worse in 23 (40.4%), while RLS patients reported themselves as better in 7 (15.6%), unchanged in 34 (75.6%), and worse in only 4 (8.9%), *p* = 0.0001]. Hypersomnolent patients who perceived their symptoms to worsen reported that symptoms had never returned to baseline in 66.7%, took months or years to return to baseline in 9.5%, and resolved in days to weeks in 23.8%. RLS patients who reported worsening of RLS reported no resolution with time in 50%, while the remaining 50% of patients reported that RLS symptoms returned to baseline within days to weeks of the procedure.

## Discussion

Anesthetics are increasingly recognized to cause long-lasting effects on the brain. In animal studies, anesthetic exposure has been associated with apoptotic brain cell death and neuronal deletion in certain parts of the brain, such as the retrosplenial cortex and thalamus (Nikizad et al., 2007; Fang et al., 2012). Long-term learning and memory impairment has also been observed in rats following anesthetic exposure (Callaway et al., 2012; Lin et al., 2014). The literature shows growing evidence for neurotoxicity in humans, such as neuroanatomical structural alterations as well as cognitive and behavioral changes in children (Lei et al., 2014; Backeljauw et al., 2015). These behavioral changes include problems with sleep (Bakri et al., 2015). A recent meta-analysis supports these findings, indicating a modestly elevated risk of neurodevelopmental disorders in children less than 3 years old following a single anesthetic exposure (Zhang et al., 2015). The long-term effects of anesthetics in geriatric populations have also been a focus practicing anesthesiologists' concern. Postoperative delirium and cognitive decline are serious potential complications experienced by the elderly adult (Hartholt et al., 2012; Murthy et al., 2015) and the middle aged adult (Johnson et al., 2002). General anesthetics may be an etiological factor in postoperative cognitive decline in elderly patients (Rasmussen et al., 2003). Our study was designed as a pilot study to explore whether anesthesia was perceived to have similarly problematic long term sequelae by patients with sleep disorders.

Indeed, our patients with central disorders of hypersomnolence frequently perceived that their sleep disorder worsened following surgery with general anesthesia. Affected subjects reported needing to sleep more hours per day and having more limitations in daily activities following their procedure. Difficulty with awakening in the morning, i.e., sleep inertia/sleep drunkenness, one of the most problematic symptoms of the hypersomnolence disorders (Trotti, 2017), was also frequently perceived to have worsened following surgery with general anesthesia among the hypersomnolent group. In contrast, patients with RLS were significantly less likely to report worsening of their disorder or of sleepiness following procedures. Because the half-life of anesthetic agents is generally short, any prolonged worsening of sleepiness post-procedure cannot easily be attributed to immediate GABA-mediated effects. Whether the putative long-term changes in hypersomnolence that we are detecting in our patients' reports may be related to changes in GABA-related neural circuitry caused by anesthetic neurotoxicity or other mechanism remains to be determined.

There are clear limitations to this study. As with any survey of past events, there is the possibility for recall and non-response biases. We could not systematically record type of anesthesia because most patients did not recall nor have access to this information, and objective measures before and after surgery with general anesthesia were not available. As anticipated from typical age of onset, the group with RLS was older than the group with hypersomnolence. We also did not have a control group without any sleep disorders. We nevertheless believe that the widespread perception among hypersomnolent patients that surgery with general anesthesia worsens their disease may represent an important clinical finding that warrants further, prospective investigation. More studies are clearly necessary to elucidate the optimal peri-operative management of patients with sleep disorders, in order to provide these patients with the safest medical care possible, in and out of the operative suite.

## Author contributions

VL, PG, DB, and LT: Conception and design, acquisition, analysis, and interpretation of data; VL and LT: Drafting the work; PG and DB: Revising critically; VL, PG, DB, and LT: Final approval; VL, PG, DB, and LT: Agreement to be accountable.

### Conflict of interest statement

LT reports funds to her institution from Balance Therapeutics and Jazz Pharma, outside the submitted work. DB reports personal fees from Ferring Pharmaceuticals, Merck, and Respicardia, outside the submitted work. The other authors declare that the research was conducted in the absence of any commercial or financial relationships that could be construed as a potential conflict of interest.

## Abbreviations

RLS: restless legs syndrome.
